# Pseudogenes Provide Evolutionary Evidence for the Competitive Endogenous RNA Hypothesis

**DOI:** 10.1093/molbev/msy183

**Published:** 2018-09-25

**Authors:** Cian Glenfield, Aoife McLysaght

**Affiliations:** Smurfit Institute of Genetics, Trinity College Dublin, Dublin, Ireland

**Keywords:** ceRNA, pseudogenes, BRAF, evolutionary rates

## Abstract

The competitive endogenous RNA (ceRNA) hypothesis is an attractively simple model to explain the biological role of many putatively functionless noncoding RNAs. Under this model, there exist transcripts in the cell whose role is to titrate out microRNAs such that the expression level of another target sequence is altered. That it is logistically possible for expression of one microRNA recognition element (MRE)-containing transcript to affect another is seen in the multiple examples of pathogenic effects of inappropriate expression of MRE-containing RNAs. However, the role, if any, of ceRNAs in normal biological processes and at physiological levels is disputed. By comparison of parent genes and pseudogenes we show, both for a specific example and genome-wide, that the pseudo-3′ untranslated regions (3′UTRs) of expressed pseudogenes are frequently retained and are under selective constraint in mammalian genomes. We found that the pseudo-3′UTR of *BRAFP1*, a previously described oncogenic ceRNA, has reduced substitutions relative to its pseudo-coding sequence, and we show sequence constraint on MREs shared between the parent gene, *BRAF*, and the pseudogene. Investigation of RNA-seq data reveals expression of *BRAFP1* in normal somatic tissues in human and in other primates, consistent with biological ceRNA functionality of this pseudogene in nonpathogenic cellular contexts. Furthermore, we find that on a genome-wide scale pseudo-3′UTRs of mammalian pseudogenes (*n* = 1,629) are under stronger selective constraint than their pseudo-coding sequence counterparts, and are more often retained and expressed. Our results suggest that many human pseudogenes, often considered nonfunctional, may have an evolutionarily constrained role, consistent with the ceRNA hypothesis.

## Introduction

The competitive endogenous RNA (ceRNA) hypothesis proposes that RNAs, expressed concurrently and with a similar complement of microRNA (miRNA) recognition elements (MREs), are capable of indirectly regulating one another by competing for a shared, limited pool of miRNA molecules (Seitz 2009; Poliseno et al. 2010; Salmena et al. 2011; Thomson and Dinger 2016). CeRNAs can be noncoding transcripts such as long noncoding RNAs (lncRNAs), circular RNAs, or expressed pseudogenes. These transcripts are capable of sequestering miRNAs that otherwise would have targeted protein-coding mRNAs, and thus reduce the amount of mRNA undergoing miRNA-mediated degradation and/or repression of translation initiation. This method of posttranscriptional regulation has been suggested as a unifying theory explaining the function of many heretofore uncharacterized expressed noncoding elements in the genome (Seitz 2009; Thomson and Dinger 2016). However, the ceRNA hypothesis remains controversial (Thomson and Dinger 2016; Smillie et al. 2018).

There is good evidence in support of ceRNA activity for specific cases. Several noncoding RNAs, mainly pseudogenes, have been shown to be oncogenic or tumor suppressive via ceRNA activity when upregulated or downregulated in various distinct cancer types. In particular, processed pseudogenes of the tumor suppressor gene *PTEN* and oncogene *BRAF*, *PTENP1* and *BRAFP1*, respectively, alter the mRNA levels of their parent genes and thus have potential tumor suppressive and oncogenic properties (Poliseno et al. 2010; Johnsson et al. 2013; Yu et al. 2014; Karreth et al. 2015). Copy number loss of *PTENP1* and copy number gain of *BRAFP1* have also been associated with their respective tumor suppressive and oncogenic potential (Poliseno et al. 2010; Karreth et al. 2015). Additional pseudogenes, such as *KRASP1*, *TUSC2P*, *OCT4P4*, *CYP4Z2P*, *GBAP1*, and *BCAS4* (a unitary pseudogene), have also been shown to exert a ceRNA effect, most often by regulating their parent gene’s expression (Poliseno et al. 2010; Marques et al. 2012; Wang, Guo, et al. 2013; Rutnam et al. 2014; Zheng et al. 2015, 2016; Straniero et al. 2017). Dysregulation of expression of these pseudogenes can thus result in pathogenic consequences.

There has been some suggestion that ceRNAs may comprise a vast network of interacting RNA transcripts, where the perturbation of one transcript in the network might have widespread knock-on effects that alter expression of a number of different transcripts (Karreth et al. 2011; Tay et al. 2011; Ala et al. 2013; Bosia et al. 2013; Bosson et al. 2014; Jens and Rajewsky 2015; Chiu et al. 2017). However, the existence of such a network and the relevance of cellular ceRNA activity in general in nonpathogenic circumstances is disputed. In particular, it is not clear that the ratio of ceRNA to miRNA expression at physiological levels is amenable to competition because it has been suggested that the number of MREs present in the transcriptome for a given miRNA far outweigh the effective number of miRNA molecules (Denzler et al. 2014, 2016).

By contrast, there are also experiments utilizing single-cell assays that demonstrate extensive ceRNA interaction (Bosia et al. 2017), as well as several examples of ceRNA activity occurring during normal cellular processes between specific transcripts. One such ceRNA is the lncRNA *lnc-mg* in mice, which regulates protein concentration of Insulin-like growth factor 2 to promote myogenesis and myogenic differentiation by competing for miRNA-125b (Zhu et al. 2017). Knockout of this lncRNA resulted in increased muscular atrophy, whereas overexpression had the opposite effect, increasing muscular hypertrophy. Additionally, during stem cell self-renewal in human, the lncRNA *linc-RoR* has been shown to regulate *OCT4*, *NANOG*, and *SOX2* by functioning as an miRNA sponge and competing with the transcripts of these genes to regulate stem cell maintenance and differentiation (Wang, Xu, et al. 2013). These studies hint at a more widespread role for ceRNAs.

A large number of pseudogenes in the human genome are expressed (at least 5–10%) (Pei et al. 2012), which creates the possibility for a widespread function as ceRNAs. An evolutionary analysis of *PTEN* pseudogenes has found multiple evolutionarily independent origins of pseudogenes across the mammalian lineage (Tang et al. 2016). The *PTENP1* pseudogene has demonstrated tumor suppressive properties in human and mouse (Poliseno et al. 2010; Poliseno and Pandolfi 2015). Intriguingly, *Heterocephalus glaber* (naked mole rat), a species famed for its longevity and resistance to developing cancer, possesses 17 *PTEN* pseudogene copies, with each copy sharing a common MRE profile in their 3′ untranslated regions (3′UTRs) (Buffenstein and Jarvis 2002; Buffenstein 2008; Seluanov et al. 2009). Pseudogenes may not always be the defunct genomic relics of gene duplication they are usually thought to be.

In contrast to *PTENP1*, overexpression of the oncogenic *BRAFP1* pseudogene contributes to the formation of B-cell lymphoma in human by acting as a ceRNA for the parent gene *BRAF*, thus increasing its protein levels (Karreth et al. 2015). *BRAFP1* is also expressed in melanoma, prostate cancer, and lung cancer cell lines, and copy number gains and transcriptional amplification of the *BRAFP1* locus are present in several additional cancer types found in The Cancer Genome Atlas (Karreth et al. 2015). However, *BRAFP1* is not expressed in primary human B cells, and whether this pseudogene performs a ceRNA function in normal tissues or developmental processes remains uncertain.

Evolutionary constraint is an unbiased arbiter of biological functionality; if a genomic element is under natural selection then it follows that that element contributes to the fitness of the organism (Doolittle 2013; Graur et al. 2013). Tests for these constraints on potential ceRNA candidates should illuminate further whether these elements have biological functions at normal physiological levels.

Here, we perform an evolutionary analysis of *BRAFP1* and find evidence for sequence constraint specifically on the 3′UTR of this pseudogene. We show conservation of specific MREs in both the coding sequence (CDS) and the 3′UTR of *BRAF* and *BRAFP1*, and we find evidence for expression of *BRAFP1* in multiple primate species and in several human tissues. We reason that pseudogenes in particular make excellent ceRNA candidates, provided they are expressed. When formed they automatically have almost identical MRE profiles as their parent genes, and therefore should make effective competitors for the same miRNAs, making these elements an ideal test set to examine conservation of ceRNA functionality. We identify previously unannotated human pseudogene orthologs across 20 mammalian genomes. We show that pseudogenes possessing an identifiable 3′UTR are more likely to be retained across these genomes and are more frequently expressed than pseudogenes without a 3′UTR. Additionally, genome-wide we see that pseudogene 3′UTR sequences are constrained, and, in particular, 3′UTRs of expressed pseudogenes exhibit the strongest constraint. Our results are suggestive of a widespread biological ceRNA function for expressed pseudogenes resulting in sequence conservation of pseudogene 3′UTRs.

## Results

### The 3′UTR of *BRAFP1* Exhibits Evolutionary Sequence Constraint in *Catarrhini* Primates

Pseudogenes of the oncogene *BRAF* are present in human (*BRAFP1*) and in mouse (*Braf-rs1*), and these processed pseudogenes are capable of functioning as oncogenic ceRNAs in both of these species. It was suggested that these two pseudogenes do not share a common origin due to the high sequence similarity between the pseudogene and the parent gene in each species and their different genomic locations (Karreth et al. 2015). We confirm that the genomic locations of *BRAFP1* and *Braf-rs1* are not syntenic, and that the pseudogenes share greater sequence similarity to the *BRAF* parent genes (88.5% and 85.9% in human and mouse, respectively) than to each other (57.8%) (supplementary fig. 1, Supplementary Material online). We also confirm that the 3′UTRs of *BRAFP1* and *Braf-rs1* are not homologous, suggesting that they have arisen via retrotransposition of different *BRAF* mRNA splice variants. No orthologs of *BRAFP1* or *Braf-rs1* (or additional *BRAF*-derived pseudogenes) could be detected in mouse or in human, respectively, suggesting independent pseudogene formation or reciprocal loss postspeciation.

**Fig. 1.**
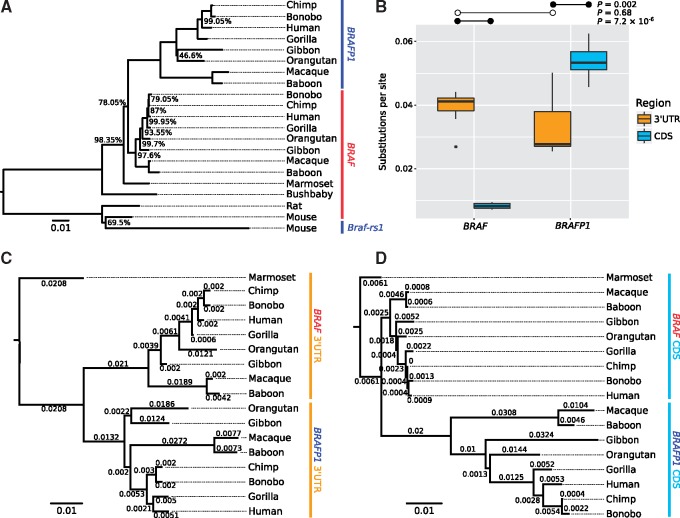
*BRAF* and *BRAFP1* phylogeny and region-specific substitution rates. (*A*) Maximum likelihood phylogeny showing the evolutionary origins of *BRAFP1* and *Braf-rs1* in primates and rodents. The phylogeny was generated from the MUSCLE (Edgar 2004) multiple sequence alignment of *BRAF* cDNA transcripts and *BRAFP1/Braf-rs1* genomic DNA from each species. Bootstrap values (2,000 replicates) are shown beside each branch where confidence is <100%. The scale is given in number of substitutions per site. (*B*) Substitutions per site for the CDS/pseudo-CDS and 3′UTR/pseudo-3′UTR are plotted for *BRAF* and *BRAFP1* from each Catarrhine species. Bonferroni-corrected *P*-values were calculated by paired *t*-test for within transcript comparisons (indicated by filled circles • in [*B*]), and Mann–Whitney *U* test for between transcripts comparison (indicated by open circles °). (*C*, *D*) Independent maximum likelihood phylogenies for the 3′UTR/pseudo-3′UTR (*C*) and CDS/pseudo-CDS (*D*), used to calculate the substitutions per site used in (*B*). Scale is in substitutions per site, and branch lengths are indicated.

To determine the timing of the origins of *BRAFP1* and *Braf-rs1*, we performed a multiple sequence alignment and maximum likelihood phylogenetic analysis of these pseudogenes and their *BRAF* parent genes (fig. 1*A*). Orthologs of *BRAFP1* were discovered in each species of the Catarrhine lineage (apes and Old World monkeys), but no other species, supporting a single origin at the base of *Catarrhini* (28–43 Ma) (Kumar et al. 2017). *Braf-rs1* could not be found in any other species tested, including rat, supporting a species-specific pseudogene origination event in mouse (<23 Ma).

That *BRAFP1* and *Braf-rs1* both have ceRNA functionality is demonstrated by the observation that an increase in their expression can be oncogenic in both species (Karreth et al. 2015). The question remains as to whether these pseudogenes have a function, ceRNA or otherwise, in normal cellular processes. To address this, we assessed the level of sequence constraint acting on *BRAFP1* in Catarrhine primates. Sequence constraint is an unbiased indicator of biological functionality (Doolittle 2013; Graur et al. 2013). Pseudogenes (and other lncRNAs) functioning as ceRNAs should be expected to show greater sequence constraint acting on their MREs, which for protein-coding genes are most commonly found in the 3′UTR, relative to the remainder of their sequence.

We performed separate phylogenetic analyses on the CDS and 3′UTR of *BRAF* and *BRAFP1* orthologs (figs. 1*C* and *D*). For convenience, we refer to the CDS or 3′UTR of the pseudogenes even though we understand these to be noncoding. Pseudo-CDS and pseudo-3′UTR are identified by sequence alignment with the parent gene. From these phylogenies, we identified the average number of substitutions per site for each CDS and 3′UTR in each species by calculating the branch lengths of each sequence back to the most recent common ancestor.

Comparing the number of substitutions between regions, we find that the *BRAFP1* 3′UTRs have a significantly lower number of substitutions relative to the CDS (Bonferroni-corrected *P *=* *0.002, paired *t*-test; fig. 1*B*). The pseudogene CDS has a higher number of substitutions than the pseudo-3′UTR and the parent gene CDS, as would be expected for a sequence under no or weaker constraint. Conversely, we find no significant difference between the number of substitutions in the *BRAF* 3′UTR and the *BRAFP1* 3′UTR (Bonferroni-corrected *P *=* *0.68, Mann–Whitney *U* test), suggesting that these sequences are under similar levels of constraint.

These observations show that the 3′UTR of *BRAFP1* is under stronger evolutionary constraint than its CDS, which ought to share the same mutation rate. This indicates that the 3′UTR of *BRAFP1* has functional properties, which is in keeping with the suggested role of this pseudogene as a ceRNA in normal cellular processes.

### MREs Associated with *BRAF* and *BRAFP1* ceRNA Activity Are Conserved

If *BRAFP1* functions as a ceRNA competing with *BRAF* for a shared pool of miRNAs, then it follows that we expect to see conservation of MREs corresponding to those miRNAs. To determine whether MRE sequence constraint is present we examined the predicted MRE profile for *BRAF* and *BRAFP1*, as well as specific MREs for previously validated miRNAs that mediate competition between these transcripts (Karreth et al. 2015). Comparing the number of shared MREs between the separate regions of these elements, that is, the number of MREs that are predicted at least once in the CDS or 3′UTR for each *BRAF* and *BRAFP1* ortholog, we find that the 3′UTR of *BRAFP1* (and *BRAF*) has a greater number of shared MREs relative to their CDS regions (*P *=* *0.002 and *P *=* *3 × 10^−^^7^, respectively; paired *t*-tests, Bonferroni-corrected; supplementary figs. 2*A* and *B*, Supplementary Material online).

**Fig. 2.**
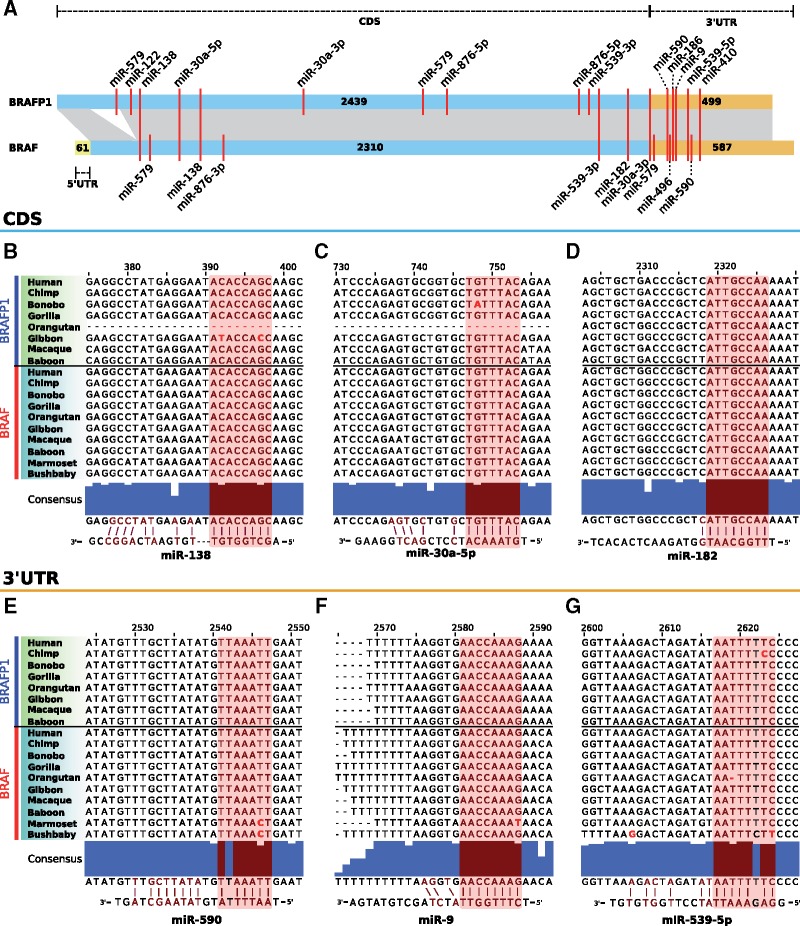
Predicted MRE locations and sequence conservation in *BRAF* and *BRAFP1*. (*A*) Locations of MREs for miRNAs confirmed and/or predicted to regulate *BRAF* and *BRAFP1* are shown with respect to the gene and pseudogene structures. MiRanda target prediction (Enright et al. 2003) was used to locate the putative MREs. Red bars spanning both elements indicate MREs present in both, whereas red bars on one element only indicate element-specific MREs. The full list of miRNAs and number of species that each MRE is predicted to be present in can be found in supplementary table 1, Supplementary Material online. Lengths of each region, corresponding to the human sequence, are indicated. Predicted conserved MREs across *BRAF* and *BRAFP1* CDS (*B–D*) and 3′UTR (*E–G*) in *Catarrhini* primates. Red shading indicates the seed region, and blue consensus bars for each base denote the proportion of sequences that match the consensus sequence. The position along sequences relative to human *BRAFP1* is indicated. The miRNA mature sequences are also shown directly below the consensus sequences. Bases in bold red font indicate substitutions predicted to be detrimental to miRNA binding. The long deletion in the pseudo-CDS of orangutan is supported by BLAST searches of two independent genome builds and inspection of intact contigs. Alignments were visualized using JalView (Waterhouse et al. 2009).

Focusing on miRNAs previously predicted or validated as regulators of *BRAF* (*n* = 17, supplementary table 1, Supplementary Material online), we found ten also have MREs in *BRAFP1* (fig. 2*A*). In particular, previous experiments have shown that mutation of the MREs for miR-30a-5p (CDS), miR-182 (CDS), and miR-590 (3′UTR) resulted in reduced ceRNA competition between *BRAF* and *BRAFP1* in cancer cell lines (Karreth et al. 2015). We find that these MREs have highly conserved sequence between the pseudogene and parent gene sequences (figs. 2*C*−*E*). In addition, we find that MREs of miRNAs that have been predicted to be regulators of *BRAF* by TargetScan (Lewis et al. 2005) and mirSRV (Betel et al. 2010) are conserved in both the parent gene and pseudogene (figs. 2*B*, *F*, and *G*;supplementary figs. 3*A*−*D*, Supplementary Material online). Specifically, MREs for miR-9 and miR-539-5p are particularly well conserved, with 100% sequence identity across *Catarrhini* for miR-9 and a single substitution in *BRAFP1* in chimp for miR-539-5p. Both of these MREs are located in the 3′UTR of these transcripts.

The greater constraint of the 3′UTR of this pseudogene may potentially reflect a greater density of conserved sites in the 3′UTR, leading to a greater proportion of its sequence exhibiting constraint. However, the spacing of these MREs indicates that those in the 3′UTR may be more important than those in the CDS. Conserved binding sites are more likely to be closer together, separated by 10–130 nucleotides, with the optimal distance deemed experimentally to be 8–40 nucleotides (Grimson et al. 2007; Sætrom et al. 2007). Proximal MREs in this manner have been shown to cooperate to enhance ceRNA activity, and fewer transcripts are required to effectively compete when this cooperation occurs (Denzler et al. 2016). The binding sites we find in the 3′UTR of *BRAF* and *BRAFP1* are within ∼40 nucleotides of each other, suggesting that these sites may be more important for effective competition between these transcripts.

### Expression of *BRAFP1* in Human Somatic and Developmental Tissues

In order for ceRNA posttranscriptional regulation to occur, both the pseudogene and its parent gene must be expressed together. Previous work examining RNA-seq data has indicated that *BRAFP1* is not expressed in normal, nonmalignant B cells (Karreth et al. 2015). This is not surprising given that overexpression of *BRAFP1* in B cells has been shown to be oncogenic. This does not, however, preclude the possibility of expression of this pseudogene in other tissues or developmental stages.

**Fig. 3.**
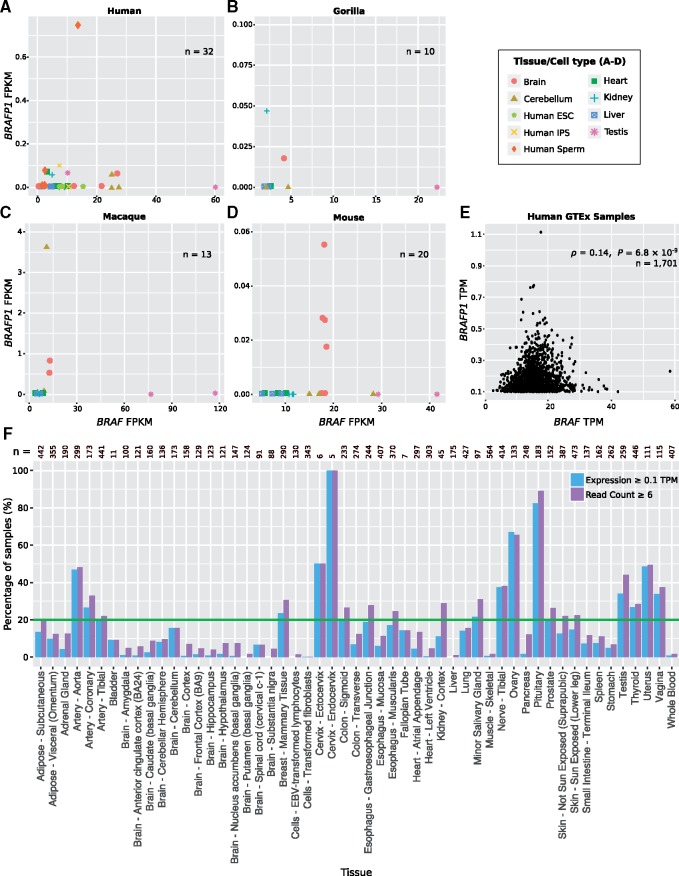
Expression of *BRAF* and *BRAFP1* in primates and mouse. (*A–D*) *BRAFP1* fragments per kilobase per million mapped reads (FPKM) levels are plotted against *BRAF* FPKM on a per sample basis. Number of samples for each species is indicated. The key (right) indicates the tissue/cell type for each sample. See Materials and Methods for RNA-seq data sources. (*E*) *BRAFP1* TPM against *BRAF* TPM levels are shown for each sample available from GTEx where *BRAFP1* expression is ≥0.1 TPM (*n* = 1,701). Samples all originate from somatic tissues. *P*-value is calculated by Spearman rank-order correlation test. (*F*) The percentage of GTEx samples in each tissue where *BRAFP1* expression is ≥0.1 TPM with ≥6 reads aligning to the pseudogene is shown. The green line at 20% indicates the threshold for expression according to GTEx criteria. The total numbers of samples from each tissue are indicated.

We first examined expression levels of *BRAF* and *BRAFP1* in RNA-seq data (see Materials and Methods) from multiple tissues in three Catarrhine species, human, gorilla, and macaque (figs. 3*A*−*C*), as well as *Braf* and *Braf-rs1* in mouse (fig. 3*D*). *BRAFP1* has low or no expression in most samples (<0.1 fragments per kilobase per million mapped reads [FPKM]), though it has relatively high expression in macaque cerebellum and brain tissues. Additionally, at least one brain tissue sample in each species exhibits expression of *BRAFP1*. By contrast, *BRAF* has a greater breadth of expression and is most highly expressed in testis in each species.

To validate these observations in a greater number of tissue samples, we utilized additional human expression data from GTEx (GTEx Consortium 2015) which includes 11,688 samples across 53 tissues. We found that *BRAF* is expressed (i.e., transcripts per million [TPM] ≥ 0.1 for a given sample) in all samples, whereas *BRAFP1* is expressed in only 1,701 samples (∼15% of the total number of samples, including samples from 49/53 tissues, fig. 3*F*). While we observe that *BRAFP1* is consistently expressed at much lower levels than its parent gene, it was previously shown that *BRAFP1* transcripts appear to be more rapidly degraded than *BRAF* transcripts (Karreth et al. 2015). Additionally, these levels are consistent with previous observations showing that *BRAFP1* is an effective ceRNA at comparably low levels (Karreth et al. 2015).

*BRAF* and *BRAFP1* expression levels are weakly correlated for those samples in which *BRAFP1* is expressed (*ρ*  =  0.14, *P *=* *6.8 × 10^−^^9^, Spearman rank-order correlation; fig. 3*E*). Though the biological significance of this observation in the context of the ceRNA hypothesis is unclear, as expression levels need not be correlated in order for a ceRNA effect to take place.

To conservatively determine in which human tissues *BRAFP1* is expressed, we follow the GTEx criteria, namely ≥0.1 TPM and ≥6 reads aligning unambiguously in at least 20% of samples for a given tissue. We found that this pseudogene is expressed in 16 of the 53 tissues based on these criteria (fig. 3*F*). As mentioned, *BRAF* levels are high in testis tissue across several species, and we can see that *BRAFP1* is also expressed in this tissue in human. Notably, none of the brain tissues examined had *BRAFP1* expression levels exceeding the GTEx threshold, despite having observed expression in the other RNA-seq data sets. Furthermore, we find almost no samples of whole blood with *BRAFP1* expression present, consistent with previous observations that it is not expressed in primary human B cells (Karreth et al. 2015). Examining additional expression data cataloged in the EBI Expression Atlas (Gerrelli et al. 2015), we found that *BRAFP1* and *BRAF* are comparatively highly expressed in human developmental brain tissues at certain stages of development (supplementary fig. 4, Supplementary Material online). In particular, *BRAFP1* is more highly expressed in these tissues (>5 TPM in choroid plexus at 10 weeks postconception) than in any samples examined previously, suggesting that this pseudogene may have an important role in embryonic brain development.

*BRAF* is an MEK kinase and its normal function as part of the RAS/MAPK pathway is to activate MEK/ERK and thus promote cellular proliferation, movement, and differentiation. *BRAF* has been shown to be necessary for extraembryonic placental development, by activating ERK and promoting vascular development (Galabova-Kovacs et al. 2006). That *BRAFP1* has high expression in several brain tissues during various stages of embryonic development, as compared with adult somatic cells, is consistent with an important role during development.

### Expressed Pseudogenes with 3′UTRs Have Retained Orthologs across Mammalian Species

**Fig. 4.**
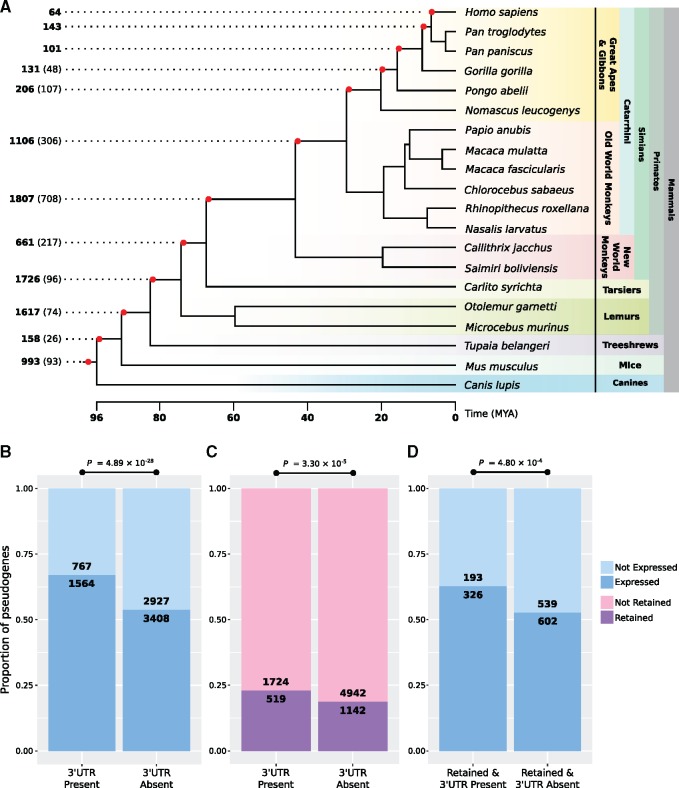
Pseudogene ortholog identification and 3′UTR retention and expression. (*A*) A time-calibrated phylogenetic tree of 20 mammalian species is shown with the number of pseudogenes originating at each branch, calculated by identifying the most distantly related species in which a pseudogene ortholog can be found. The numbers in brackets indicate the number of retained pseudogenes originating at that branch, that is, the number of pseudogenes which have an ortholog present in each descendant from that point. Time calibrations were obtained from TimeTree (Kumar et al. 2017). (*B*) Barplot showing the proportion of expressed pseudogenes (median TPM ≥0.1 in at least one tissue) with an identifiable 3′UTR compared with the proportion where a 3′UTR is not present (i.e., could not be identified). (*C*) Barplot showing the proportion of fully retained pseudogenes (minimum of 5 species) with a 3′UTR compared with those without a 3′UTR. (*D*) Barplot showing the proportion of expressed pseudogenes that are retained and also have a 3′UTR present, compared with those that are retained but do not have a 3′UTR. Bonferroni-corrected *P*-values were calculated by χ^2^ test.

Our finding of sequence constraint in *BRAFP1* raises the question as to whether there is a genome-wide trend of pseudogene sequence conservation consistent with a broad role for ceRNAs. We analyzed pseudogene–parent gene pairs from the GENCODE Pseudogene Decoration Resource (PsiDR) (Pei et al. 2012). We identified orthologs of 8,704 human pseudogenes across 20 mammalian species by extracting genomic sequences syntenic to human pseudogene annotations from a 20-way high-quality whole-genome multiple sequence alignment downloaded from the UCSC Genome Browser (Kent et al. 2002). The age of each pseudogene was estimated by ascertaining the most distantly related species for which an ortholog can be discovered (fig. 4*A*). We also examined patterns of pseudogene retention across these genomes, and we define a pseudogene as “retained” if it is present in each descendant of the branch where we infer origin. In total, we consider 1,674 (∼19.2%) pseudogenes to be retained (fig. 4*A* and table 1). Notably, 93 pseudogenes (∼9.3%) of the 993 that originated at or before the oldest branching point—the divergence with canines—are retained and present across all 20 species. Additionally, we find that processed pseudogenes are more likely to be retained than duplicated pseudogenes (Bonferroni-corrected *P *=* *1.34 × 10^−^^34^, χ^2^ test; table 1).

**Table 1.** Numbers of Retained and 3′UTR-Containing Pseudogenes Expressed in Human.

	Total Set	Retained	Not Retained	3′UTR Present	3′UTR Absent	Retained + 3′UTR	Retained − 3′UTR
**Expressed (≥0.1 TPM)**^a^	4,944	928	3,824	1,564	3,380	326	602
**Not expressed (<0.1 TPM)**^a^	3,760	746	2,899	767	2,993	193	539
**Total No.**	8,704	1,674	6,723	2,331	6,373	519	1,141
**All median TPM**^a^	0.1545	0.1311	0.1566	0.2660	0.1291	0.1878	0.1122
**All *P*-value**^b^	—	0.015		4.86 × 10^−35^		6.10 × 10^−7^	
**Expressed only median TPM**^a^	0.6070	0.4732	0.6332	0.6647	0.5919	0.5289	0.4581
**Expressed only *P*-value**^b^	—	4.03 × 10^−7^		0.18		1	
**Processed**	7,364	1,605	5,603	2,101	5,263	508	1,097
**Duplicated**	1,195	61	984	226	969	11	50
**Total No.**	8,559	1,666	6,587	2,327	6,232	519	1,147

a Median expression, from GTEx data, in the most highly expressed tissue per pseudogene.

b Bonferroni-corrected *P*-values for all (including nonexpressed) and expressed only pseudogenes calculated by Mann−Whitney *U* test.

Given the observation of sequence constraint in the pseudo-3′UTR of *BRAFP1*, we wanted to test for constraint of this region genome-wide. As pseudogene 3′UTRs are not annotated, we identified these regions by querying the 3′UTRs of the parent genes against their respective pseudogenes using BlastN. For duplicated pseudogenes, which also contain pseudointrons, we queried the parent gene’s CDS regions against these to obtain the pseudogene’s CDS regions. We find that identification of 3′UTRs was not biased toward younger pseudogenes, which might be expected if these elements are not under constraint. We identify 3′UTRs in 2,331 pseudogenes using this method (table 1), and we find that processed pseudogenes are more likely to have an identifiable 3′UTR than duplicated pseudogenes (Bonferroni-corrected *P *=* *1.06 × 10^−^^11^, χ^2^ test).

We define a pseudogene as expressed if it has a median TPM of ≥0.1 in at least one tissue. This expression cutoff minimizes false positives and false negatives (supplementary fig. 5, Supplementary Material online). In total, we define 4,944 (∼57%) pseudogenes as expressed in at least one human tissue (table 1), a much higher proportion than previously reported (Pei et al. 2012). Those pseudogenes that possess a 3′UTR are more highly expressed when considering the total set of pseudogenes (Bonferroni-corrected *P *=* *4.86 × 10^−^^35^, Mann–Whitney *U* test) but not the expressed only set (Bonferroni-corrected *P *=* *0.18, Mann–Whitney *U* test). Importantly, however, we find that pseudogenes possessing a 3′UTR are both more likely to be expressed (Bonferroni-corrected *P *=* *4.89 × 10^−^^28^, χ^2^ test; fig. 4*B*) and retained (Bonferroni-corrected *P *=* *3.30 × 10^−^^5^, χ^2^ test; fig. 4*C*) than those without a 3′UTR. In addition, while retained pseudogenes are not more likely to be expressed than those not retained (Bonferroni-corrected *P *=* *1, χ^2^ test; table 1), pseudogenes that both are retained and have a 3′UTR are more likely to be expressed than those that are retained but do not have a 3′UTR (Bonferroni-corrected *P *=* *4.80 × 10^−^^4^, χ^2^ test; fig. 4*D*).

### Pseudogene 3′UTRs Are under Stronger Evolutionary Constraint

**Fig. 5.**
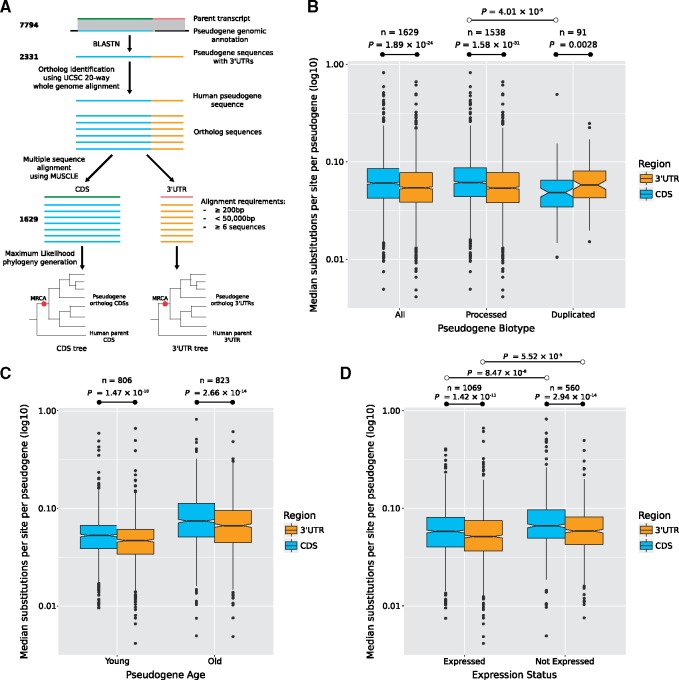
Constraint of 3′UTRs across pseudogene orthologs. (*A*) Pseudogene 3′UTRs were identified using BlastN (Altschul et al. 1990), and pseudogene orthologs were previously identified by extracting syntenic regions in a whole-genome alignment of 20 mammalian genomes. The pseudogene CDS regions and 3′UTRs were separated and aligned for each pseudogene, with subsequent phylogeny generation. Median branch lengths (i.e., substitutions per site) to the most recent common ancestor (MRCA, red dot) were then calculated for each pseudogene CDS and 3′UTR pair. The number of pseudogenes passing each stage of the work flow are indicated on the left. (*B*) Box and whisker plot showing the distribution of median substitutions per site of the CDS and 3′UTR, for the total set of pseudogenes (processed and duplicated) as well as separated by pseudogene biotype. (*C*) Box and whisker plot showing the distribution of median substitutions per site of CDS and 3′UTR separated by young or old pseudogenes. Young pseudogenes originated on branches at or after the divergence of simians from tarsiers, and old pseudogenes are those that originated at branches before this divergence (see fig. 4*A*). (*D*) Box and whisker plot showing the distribution of median substitutions per site for the CDS and 3′UTR separated by pseudogene expression status, either expressed (median TPM ≥ 0.1) or not expressed (median TPM < 0.1). Bonferroni-corrected *P*-values were calculated by Wilcoxon signed-rank test (•) or by Mann–Whitney *U* test (°).

To examine sequence constraint in a similar manner to *BRAF* and *BRAFP1*, we limited our pseudogene set to only those that had CDS regions and 3′UTRs that both pass our filtering criteria (*n* = 1,629, fig. 5*A*). When we compared the median number of substitutions per site between the CDS regions and 3′UTRs (see Materials and Methods) of the total set of pseudogenes, we found that the 3′UTRs have significantly fewer substitutions than their CDS regions (Bonferroni-corrected *P *=* *1.89 × 10^−^^24^, Wilcoxon signed-rank test; fig. 5*B*), mirroring our findings for *BRAFP1*. This is true for both young (*n* = 806, originated on branch 7 or younger, at the base of the simian lineage) and old (*n* = 823) pseudogenes (Bonferroni-corrected *P *=* *1.47 × 10^−^^10^ and *P *=* *2.66 × 10^−^^14^, respectively, Wilcoxon signed-rank tests; fig. 5*C*), suggesting that this trend is not biased by pseudogene age.

By contrast, a similar analysis of parent genes (*n* = 274) shows that their CDS regions have significantly fewer substitutions relative to their 3′UTRs, which is expected of protein-coding genes (Bonferroni-corrected *P *=* *2.7 × 10^−^^41^, Wilcoxon signed-rank test; supplementary fig. 6, Supplementary Material online). Notably, when we restrict the pseudogene analysis to the duplicated (*n* = 91) pseudogenes (as distinct from retropseudogenes), we observe that, unlike the general trend for pseudogenes, the CDS regions have fewer substitutions (*P *=* *0.0028, Wilcoxon signed-rank test; fig. 5*B*). Additionally, the CDS regions of duplicated pseudogenes have fewer substitutions than the corresponding regions of processed pseudogenes (Bonferroni-corrected *P *=* *4.01 × 10^−^^5^, Mann–Whitney *U* test). Young DNA-based duplicates have expression patterns that closely mirror the parent gene (Guschanski et al. 2017). Even though the genes we analyzed are annotated as pseudogenes in human, those that originated by DNA-based duplication may have retained functionality (and thus evolutionary constraint) in some nonhuman species or for an initial period postduplication. Such a scenario would consequently reduce the overall median number of substitutions for their CDS regions.

We found that the median number of substitutions in the 3′UTRs is significantly lower than their CDS regions for both expressed and nonexpressed pseudogenes (Bonferroni-corrected *P *=* *1.42 × 10^−^^11^ and *P *=* *2.94 × 10^−^^14^, respectively, Wilcoxon signed-rank tests; fig. 5*D*). Overall, the 3′UTRs of the expressed pseudogenes have the lowest median number of substitutions per site among each of these groups (Bonferroni-corrected *P *=* *5.52 × 10^−^^5^, Mann–Whitney *U* test). Additionally, we examined expression correlation between pseudogene–parent gene pairs in tissues where both are expressed. We found that duplicated pseudogenes were more likely to show correlated expression with their parents than processed pseudogenes (Bonferroni-corrected *P *=* *1.10 × 10^−^^12^, χ^2^ test), though the biological significance of this, if any, is not obvious. Pseudogenes with correlated expression were not more likely to possess 3′UTRs (Bonferroni-corrected *P *=* *0.3, χ^2^ test) and were less likely to be retained (Bonferroni-corrected *P *=* *0.0003, χ^2^ test).

Of the expressed pseudogenes tested (*n* = 1,061), we found that pseudogenes with both correlated and noncorrelated expression have significantly fewer substitutions in their 3′UTRs (Bonferroni-corrected *P *=* *4.89 × 10^−^^5^ and *P *=* *3.08 × 10^−^^8^, respectively, Wilcoxon signed-rank tests; supplementary fig. 7*A*, Supplementary Material online). The CDS regions and 3′UTRs have fewer substitutions in expression-correlated pseudogenes than in their noncorrelated counterparts (Bonferroni-corrected *P *=* *5.65 × 10^−^^7^ and *P *=* *9.68 × 10^−^^5^ for CDS and 3′UTR comparisons, respectively, Mann–Whitney *U* tests). Separating the correlated pseudogenes into positively or negatively correlated, we found that both of these groups showed fewer 3′UTR substitutions (Bonferroni-corrected *P *=* *0.014 and *P *=* *0.0012, respectively, Wilcoxon signed-rank tests; supplementary fig. 7*B*, Supplementary Material online) and the CDS regions of positively correlated pseudogenes have fewer substitutions than that of negatively correlated pseudogenes (Bonferroni-corrected *P *=* *0.0068, Mann–Whitney *U* test). However, we found that the 3′UTRs were not significantly different between positively and negatively correlated pseudogenes (Bonferroni-corrected *P *=* *1, Mann–Whitney *U* test), though the median number of substitutions for pseudogenes that have positively correlated expression with their parents is lower than all other groups tested.

Finally, we tested pseudogene loci for signatures of recent positive selection (i.e., within human populations) using precomputed statistical measures from dbPSHP on single nucleotide polymorphism (SNP) data from the 1000 Genomes Project (Li et al. 2014; Auton et al. 2015). We extracted data on Tajima’s *D*, fixation index (*F*_ST_), derived allele frequency difference (ΔDAF), and the cross-population composite likelihood ratio (XP-CLR) from this study. We found that 4,741 (54.5%) of 8,693 pseudogene loci contain at least one SNP in at least one population that passes one or more score threshold (Li et al. 2014). More conservatively, 503 pseudogenes pass positive selection thresholds in at least three of the tests, and 22 pseudogenes pass all four, the latter being the most confident inferences (supplementary tables 2 and 3, Supplementary Material online). We found that 11 (50%) of the top 22 potentially positively selected pseudogenes possesses an identifiable 3′UTR, compared with only ∼26.8% of the total set of pseudogenes (*P *=* *0.026, Fisher’s exact test). Additionally, 6 (∼27.3%) of these top pseudogenes are retained across species, compared with ∼20.2% for the total set (*P *=* *0.42, Fisher’s exact test).

## Discussion

There has been substantial interest in the ceRNA hypothesis in recent years, with much of the research in the area revolving around how dysregulation of ceRNA expression can affect cancer pathogenicity and progression (Poliseno et al. 2010; Karreth et al. 2011, 2015; Poliseno and Pandolfi 2015). While some studies have demonstrated the effects of certain ceRNAs in normal tissues and cells, for example, *lnc-mg* and *linc-RoR* (Wang, Xu, et al. 2013; Zhu et al. 2017), examples of ceRNA function at normal physiological levels are lacking. In particular, some studies dispute that this function could be widespread in normal tissues, claiming that any bona fide ceRNAs found would be exceptional cases only (Denzler et al. 2014, 2016). However, these studies focused on a small number of very highly expressed miRNAs, which are perhaps least amenable to ceRNA activity as the miRNA abundance is unlikely to be limiting (Chang et al. 2004).

Testing for evolutionary constraint of ceRNAs gives us an unbiased indicator of biological functionality; if a genomic element displays hallmarks of natural selection, then it follows that that element has contributed to the fitness of the organism. Importantly, natural selection is sensitive to fitness advantages more subtle than can readily be detected in an experimental setup. Modeling of ceRNA activity suggests that the greater the number of miRNAs mediating competition between two RNA transcripts, the more likely it is that the expression changes experienced by these transcripts are physiologically relevant (Chiu et al. 2018). Considering these findings it is clear that pseudogenes in particular make excellent ceRNA candidates, provided they are expressed, since when formed they automatically have identical MRE profiles to their parent genes, and therefore should make effective competitors for the same miRNAs.

In general, 3′UTRs of protein-coding genes are under sequence constraint in mammals, though at a much lower level than their CDS regions (Davydov et al. 2010). Our finding that the 3′UTR of *BRAFP1* is under greater sequence constraint than its CDS is therefore interesting. Given that the majority of currently validated MREs reside within the 3′UTR region of protein-coding genes, the fact that we see greater constraint of this region in *BRAFP1* is consistent with conservation of miRNA-binding sites for this pseudogene. Furthermore, it was recently shown that transcripts acting as ceRNAs for tumor suppressor genes are enriched for 3′UTR shortening via alternative polyadenylation in breast cancer tumors, disrupting ceRNA crosstalk and repressing tumor suppressor gene activity in trans (Xia et al. 2014; Park et al. 2018). This finding further illustrates the importance of 3′UTRs to ceRNA regulation.

Our observation of increased constraint on the 3′UTR of *BRAFP1* is mirrored genome-wide, and we find that pseudogenes with 3′UTRs are more likely to be expressed and retained. However, our genome-wide expression analyses were limited to human GTEx data. An investigation of pseudogene expression in other lineages may exhibit stronger or weaker correlations between expression and 3′UTR retention. Interestingly, we find tentative evidence for recent positive selection of some pseudogene loci in human. Considering many currently validated ceRNA interactions occur between pseudogenes and their respective parent genes (Poliseno et al. 2010; Wang, Guo, et al. 2013; Rutnam et al. 2014; Zheng et al. 2015, 2016; Straniero et al. 2017), our finding of pseudogene constraint over evolutionary timescales suggests a widespread ceRNA role for pseudogenes.

## Materials and Methods

### *BRAFP1* and *Braf-rs1* Evolutionary Origins, Phylogenetic Reconstruction, and Region-Specific Substitution Analysis

Human *BRAF* (ENSG00000157764) and mouse *Braf* (ENSMUSG00000002413) cDNA and human *BRAFP1* (ENSG00000224775) genomic sequences were retrieved from Ensembl (GRCh38.p7 and GRCm38.p5) (Aken et al. 2017). As *Braf-rs1* is unannotated in mouse, this sequence was identified in the mouse genome using BlastN with *Braf* as a query (Altschul et al. 1990). Pairwise comparisons between *BRAF, Braf, BRAFP1*, and *Braf-rs1* were conducted using Emboss Needle pairwise sequence alignment (Rice et al. 2000). As these pseudogenes are not annotated in other species, *BRAFP1* and *Braf-rs1* sequences were used to query reference genomes in additional primate and rodent species using BlastN with default parameters to identify orthologous sequences. Potential orthologs were verified by manual inspection for conserved gene order flanking the hits. The ortholog sequences were obtained from GenBank (Benson et al. 2012).

To reconstruct the evolutionary origins of these pseudogenes, *BRAF* and *Braf* cDNA sequences and *BRAFP1* and *Braf-rs1* genomic sequences were aligned using MUSCLE multiple sequence alignment (Edgar 2004). Rat, marmoset and bushbaby *BRAF* cDNA sequences were used as outgroups for mouse and Catarrhine primates, respectively, as *Braf-rs1* or *BRAFP1* orthologs were not found in these species. In addition, *Catarrhini BRAFP1* and *BRAF*, including marmoset *BRAF*, sequences were separated into (pseudo-)CDS regions and (pseudo-)3′UTRs, which were subsequently aligned separately. The resulting phylogenies were inferred using the maximum likelihood method based on the Tamura three-parameter (T92) model (Tamura 1992). This was determined to be the best model for these data based on the Bayesian Information Criterion score calculated for each model using MEGA7 (Kumar et al. 2016). Phylogenies constructed using the Tamura–Nei (TN93) model are also consistent with those from T92 (Tamura and Nei 1993). The trees are drawn to scale with branch lengths measured in number of substitutions per site. For each alignment, all positions with <60% site coverage were eliminated (i.e., positions in the alignments where there were gaps in more than 40% of the sequences). To test each phylogeny, the bootstrap method with 2,000 replicates was used. These evolutionary analyses were conducted in MEGA7 (Kumar et al. 2016), and phylogenetic tree figures were produced in FigTree v1.4.3 (Rambaut 2016).

Region-specific numbers of substitutions per site were obtained by calculating the distance, or branch length, of each sequence to the most recent common ancestor of *BRAF* and *BRAFP1*.

### Prediction of MREs and Analysis of Validated *BRAF* and *BRAFP1* miRNA Interactions

Supplementary table 1, Supplementary Material online, lists miRNAs experimentally validated or predicted to bind to *BRAF* and/or *BRAFP1* in human, identified from various sources. Mature sequences for these miRNAs were obtained from http://www.mirbase.org/ (Kozomara and Griffiths-Jones 2014), and locations of MREs for these miRNAs in *BRAF* and *BRAFP1* across Catarrhine species were predicted using miRanda target prediction software (Enright et al. 2003). The multiple sequence alignments with consensus sequences, shown in figures 2*B*−*G* and supplementary figures 3*A−D*, Supplementary Material online, were visualized using JalView (Waterhouse et al. 2009).

In addition, a total set of 2,588 human mature miRNA sequences were obtained from miRBase 21 (Kozomara and Griffiths-Jones 2014). Also, 23,000 genomic sequences of 3,000 bp each from randomized genomic locations, 1,000 sequences from each human chromosome, were generated. MREs for each of these sequences were predicted using miRanda with the full set of human miRNAs to estimate the genomic density of spurious MREs per 100 bp (supplementary fig. 2, Supplementary Material online). Predicted MRE density was also calculated in a similar fashion for each *BRAF* and *BRAFP1* sequence across Catarrhine species (marmoset *BRAF* was also included), with further separation into CDS regions and 3′UTRs.

### Analysis of *BRAF* and *BRAFP1* Expression

RNA-seq data for human, gorilla, macaque, and mouse tissues were retrieved from multiple studies with publicly available data in the NCBI Gene Expression Omnibus database (GSE30352, GSE50781, and GSE57096) (Brawand et al. 2011; Hammoud et al. 2014; Wunderlich et al. 2014). From these data sets, *BRAF* and *BRAFP1* FPKM values for each sample were evaluated using the Tuxedo suite of tools with standard parameters (Tophat, Bowtie, Cufflinks, and Cummerbund) (Trapnell et al. 2012). As the pseudogenes are not annotated in nonhuman species, *BRAFP1* locations in the gorilla and macaque genomes and *Braf-rs1* in the mouse genome were manually annotated in their respective species’ GTF files to facilitate mapping of *BRAFP1* and *Braf-rs1* RNA reads. Additional data for human *BRAF* and *BRAFP1* for 11,688 samples comprising 53 tissues were retrieved from the GTEx Consortium database (GTEx Consortium 2015). RNA-seq data for *BRAF* and *BRAFP1* expression shown in supplementary figure 4, Supplementary Material online, were obtained from Gerrelli et al. (2015). Dot plots were generated in R using ggplot2, and the Spearman correlation analysis was performed using the Python scipy stats package (Oliphant 2007; Wilkinson 2011; R Development Core Team 2016).

### Identifying Accurate Human Pseudogene Coordinates

PsiDR has annotated the most likely parent gene and parent transcripts for 9,052 human pseudogenes (Pei et al. 2012). Gene IDs and genomic locations for each of these pseudogenes were obtained from Ensembl using the transcript IDs provided by PsiDR. Parent gene cDNA sequences were also obtained using the parent gene transcript IDs. To ensure accurate and full pseudogene coordinates, as many current pseudogene annotations do not include the pseudo-3′UTR, parent gene cDNA sequences were queried against the human reference genome (GRCh38) using BlastN. If the BLAST hit for a given parent gene overlapped with their respective pseudogene, any extension on either flank, with up to a 100-bp gap allowed, was added to the existing pseudogene coordinates. A total of 4,084 pseudogene annotations were extended using this method, and these new coordinates were used alongside existing pseudogene coordinates where no extended overlap was detected. Pseudogene genomic sequences using these updated coordinates were obtained from Ensembl (Aken et al. 2017).

### Identifying Human Pseudogene Orthologs in Mammalian Species

A high-quality hg38 20-way mammalian whole-genome alignment was downloaded from UCSC in MAF format (http://hgdownload.cse.ucsc.edu/goldenpath/hg38/multiz20way/). Using the updated pseudogene coordinates, and limiting our analyses to pseudogenes with parent genes identified, we used the mafsinRegion tool provided by UCSC (hgdownload.cse.ucsc.edu/admin/exe/linux.x86_64/mafsInRegion) to extract syntenic orthologous pseudogene sequences across these 20 mammalian species. As this alignment is in MAF format each pseudogene ortholog was extracted in a series of alignment blocks, which were subsequently concatenated to generate full-length pseudogene ortholog sequences. We also do this for the parent gene sequences, to ensure homologous parent genes are not incorrectly identified as pseudogene orthologs. Furthermore, to ensure accurate identification, only orthologs with a length of 50% or more of the human pseudogene sequence were considered as an ortholog. We identify mammalian orthologs (or lack thereof) for 8,704 human pseudogenes across these 20 species.

### Determination of Human Pseudogene Retention and Expression

The age of origination of each pseudogene was determined by identifying the most distantly related species for which a pseudogene ortholog can be found. The branch of origin of the pseudogene is inferred to be the branch leading to the last common ancestor of all species where the pseudogene is present (Dollo parsimony). A pseudogene is considered “retained” if it is present in every species descending from the branch of origin. This retention analysis was only performed for pseudogenes with presence/absence information in at least five species (i.e., pseudogenes originating on the fourth branching point from human or older).

Human pseudogene expression data were obtained from the GTEx Consortium median TPM per tissue data file (version 7), and a pseudogene is considered expressed if the median TPM ≥0.1 in its most highly expressed tissue (GTEx Consortium 2015). Expression correlations between pseudogene–parent gene pairs were calculated by Spearman rank correlation, Bonferroni-corrected for multiple testing. Only pairs where the pseudogene and parent gene are both expressed in at least one tissue were considered (*n* = 4,779). All samples from each tissue where the pair is expressed were pooled for correlation testing.

Pseudogene biotypes, either processed or duplicated (nonprocessed), were determined based on PsiDR annotations (Pei et al. 2012).

### Analysis of Pseudogene CDS and 3′UTR Substitution Differences

To identify the locations of pseudo-3′UTRs for each pseudogene, 3′UTR sequences from associated parent gene transcripts were queried against pseudogene sequences using BlastN and the resulting 3′UTR start coordinate at the 3′-end was identified. 3′UTRs for 2,331 pseudogenes were detected using this method (3′UTR present), with the rest of the pseudogenes considered as having no identifiable 3′UTR (3′UTR absent). For each processed pseudogene with a 3′UTR, the CDS and 3′UTR sequences were separated according to the 3′UTR start coordinate identified. For duplicated human pseudogenes and their orthologs, the sequence regions corresponding to the CDS of their parent genes were identified by querying the human parent gene CDS against their respective pseudogenes. The resulting hits were joined to form a pseudo-CDS sequence for each duplicated pseudogene and its orthologs.

In order to ensure accurate alignment of these orthologous CDS regions and 3′UTRs, these sequences were filtered according to the following criteria: Must be ≥200 and ≤50,000 bp in each region in each species, and must be present in at least five species including human. Additionally, using Ensembl gene annotations (GRCh38) we excluded pseudogenes with potentially functional overlapping loci on the opposite strand to ensure these loci did not confound the results (Aken et al. 2017). After filtering there were 1,629 pseudogenes that each had at least six (including human parent gene outgroup) orthologous sequences for both the CDS regions and the 3′UTRs. These sequences were subsequently aligned using MUSCLE. We tested these alignments for the best-fitting maximum likelihood phylogenetic model for all sites, ranked based on the Bayesian Information Criterion score, using the Find Best Model function in MEGA Compute-Core (Kumar et al. 2012, 2016). The Tamura three-parameter model (T92) was most frequently the best model based on our sequence data, and we therefore used this model in our subsequent analyses. We also tested other models such as the Tamura–Nei model (TN93), T2 + Gamma distribution, and the Kimura two-parameter model (K2) (Kimura 1980; Tamura 1992; Tamura and Nei 1993). Our results were consistent across each of these models. In total, 3,430 phylogenies were generated. The Dendropy package for python was used to analyze the resulting phylogenies and calculate the distance, in number of substitutions, from each pseudogene ortholog sequence to the most recent common ancestor of the pseudogene (Sukumaran and Holder 2010). Each pseudogene thus has a single median value for its CDS and 3′UTR, corresponding to the median branch length to the most recent common ancestor of the pseudogene orthologs. These values were then used in the subsequent analyses.

As a positive control, a similar analysis was performed for the CDS regions and 3′UTRs of parent genes of these pseudogenes (*n* = 274) using the T92 model. Parent genes with one-to-one orthologs in at least five of the species since pseudogene formation and with ≥200 bp in each region were used for the analysis, with the corresponding human pseudogene regions used as the outgroups.

### Positive Selection

We obtained precomputed positive selection statistical measures from the dbPSHP database of recent positive selection in human populations (http://jjwanglab.org/dbpshp), based on SNP data from the 1000 Genomes Project (http://www.internationalgenome.org/data) (Li et al. 2014; Auton et al. 2015). Pseudogene coordinates were converted to GRCh37 to match this database using UCSC LiftOver (Kent et al. 2002). Four different selection measures were used; Tajima’s *D*, ΔDAF, XP-CLR, and *F*_ST._ Pseudogenes were selected based on whether they contain SNPs with any of these measures exceeding their threshold scores; Tajima’s *D* < 0, ΔDAF > 0.2, XP-CLR > 5, and *F*_ST_ > 0.05 (Li et al. 2014).

### Statistical Analyses

The paired *t*-tests for the *BRAF* and *BRAFP1* substitution analyses and shared MRE analysis were performed by pairing values from the CDS and 3′UTR of each pseudogene. We consider these dependent values because the regions are located next to one another in the genome and are unlikely to be completely independent, with the null hypothesis being that no difference exists in the substitution rates or number of shared MREs between these regions. Similarly, for each Wilcoxon signed-rank test between the median number of substitutions of the CDS regions and 3′UTRs across the genome, we pair the median CDS and 3′UTR value of each pseudogene or parent gene and then compare the overall CDS and 3′UTR distributions. *P*-values are Bonferroni-corrected where appropriate. Statistical analyses were performed in R (3.3.1) or in the Python scipy stats package (version 1.0.0) (Oliphant 2007; R Development Core Team 2016).

## Supplementary Material

Supplementary data are available at *Molecular Biology and Evolution* online.

## Supplementary Material

Supplementary Data
