# Supplementary material for: Pseudogenes Provide Evolutionary Evidence for the Competitive Endogenous RNA Hypothesis
Source: Mol Biol Evol. 2018 Sep 25;35(12):2886–99. doi: 10.1093/molbev/msy183 (PMC6278865; doi:10.1093/molbev/msy183)
Supplement: Supplementary Data [file msy183_supp.zip › supplementary_figures.pdf]

Supplementary Information

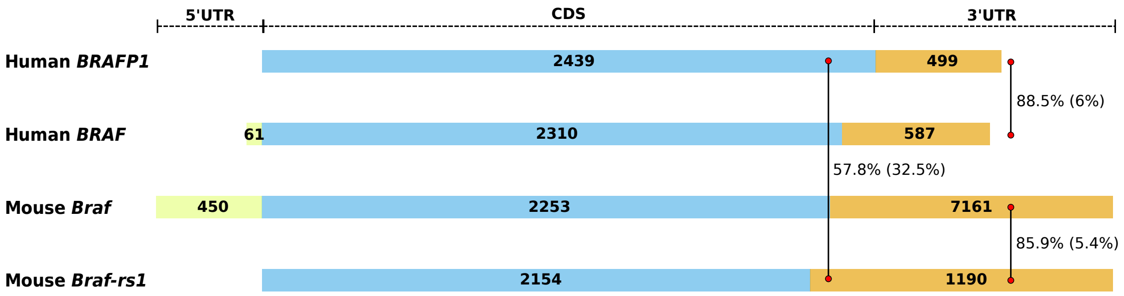

Supplementary Figure 1. Pairwise sequence similarity between human and mouse *BRAF* homologs. Overview of pairwise sequence alignments using Needle (Rice et al. 2000) between *BRAF* and *BRAFP1* in human and *Braf* and *Braf-rs1* in mouse. Linkers between sequences indicate sequence similarity comparisons. Grey shading outlines homologous sequences.

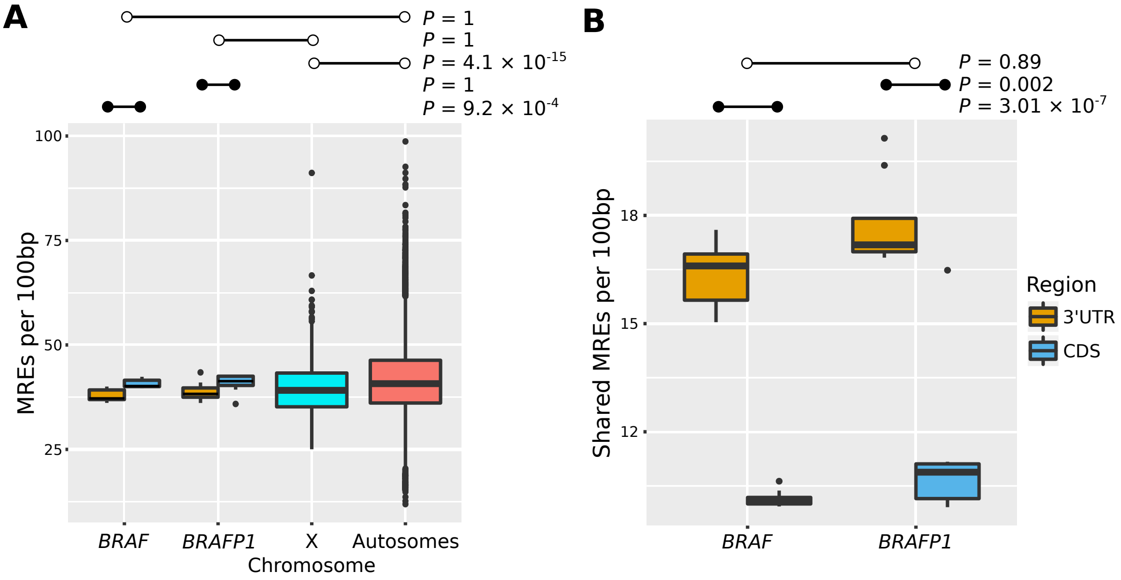

Supplementary Figure 2. MRE density across *BRAF* and *BRAFP1* sequences. **(A)** MRE density per 100bp of *BRAF* and *BRAFP1* by region for all human miRNAs is plotted with the MRE density of random sequences for the X-chromosome and autosomes. Bonferroni-corrected *P*-values for comparisons between the CDS and 3'UTR of *BRAF* and *BRAFP1* individually are by Paired t-test (•) and for between *BRAF* and *BRAFP1*, and random autosomal and X-chromosome sequence, by Mann-Whitney U test (○). **(B)** Shared MRE density per 100bp is plotted for separate regions of *BRAF* and *BRAFP1*. Each parent and pseudogene region has a single value for the number of shared MREs per 100bp. MREs are considered shared if the corresponding miRNA is predicted to bind at least once to each *BRAF* and *BRAFP1* sequence, including a related outgroup *BRAF* sequence (marmoset). Bonferroni-corrected *P*-values for comparisons between the CDS/pseudo-CDS and 3'UTR/pseudo-3'UTR of *BRAF* and *BRAFP1* individually are by Paired t-test (•) and for between *BRAF* and *BRAFP1* by Mann-Whitney U test (○).

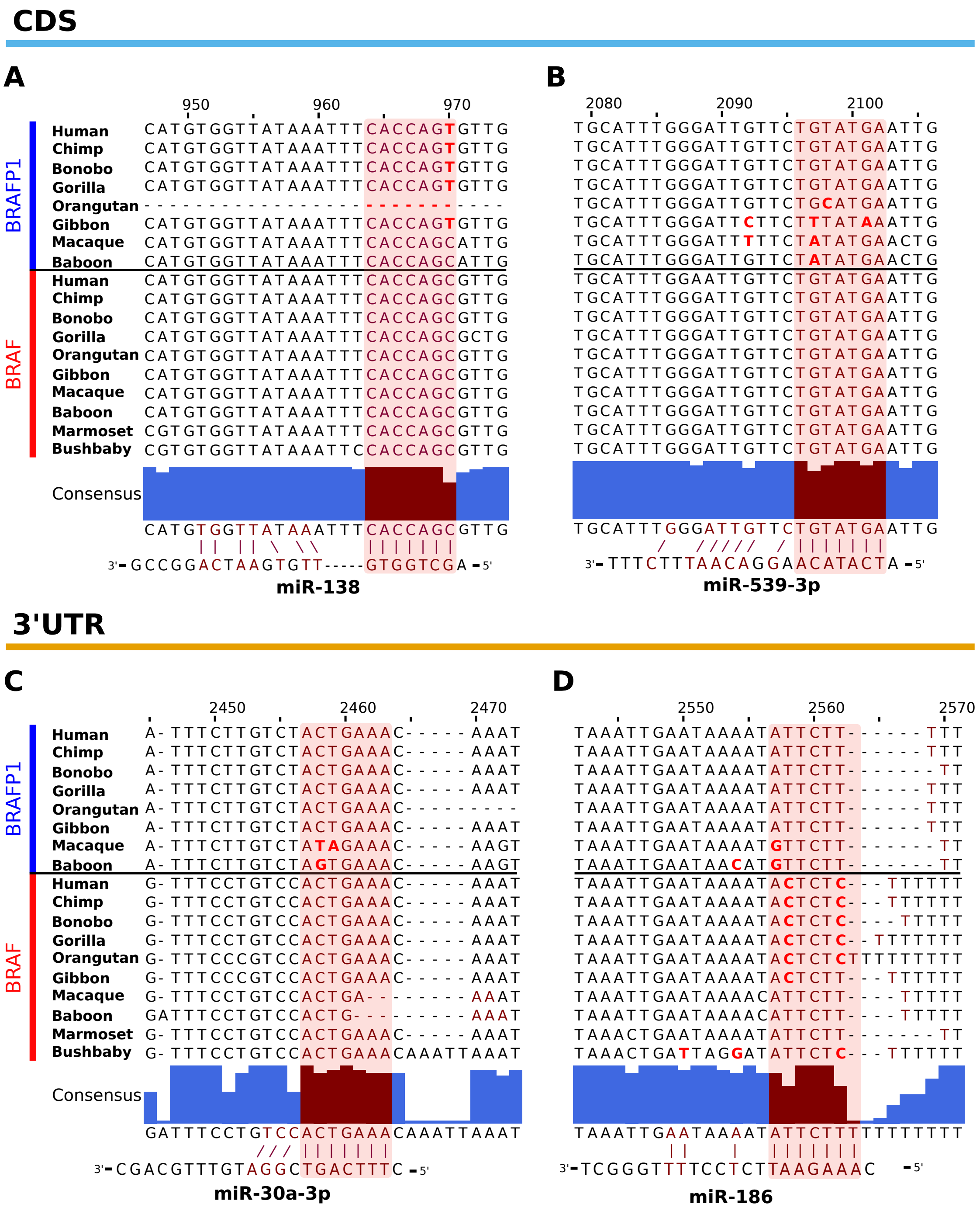

Supplementary Figure 3. Alignments of additional predicted MREs across *BRAF* and *BRAFP1*. **(A-D)** MREs predicted to bind to both *BRAF* and *BRAFP1* in at least one species are shown for the CDS **(A,B)** and 3'UTR **(C,D)**. Bold red bases indicate mutations predicted to abrogate miRNA binding for the shown miRNAs. The red shaded regions indicate the MRE seed regions. The consensus sequence along with the proportions of bases matching this sequence (blue bars) are shown beneath each alignment. The long deletion in the pseudo-CDS of orangutan is supported by BLAST searches of two independent genome builds and inspection of intact contigs. Alignments were visualised using JalView (Waterhouse et al. 2009).

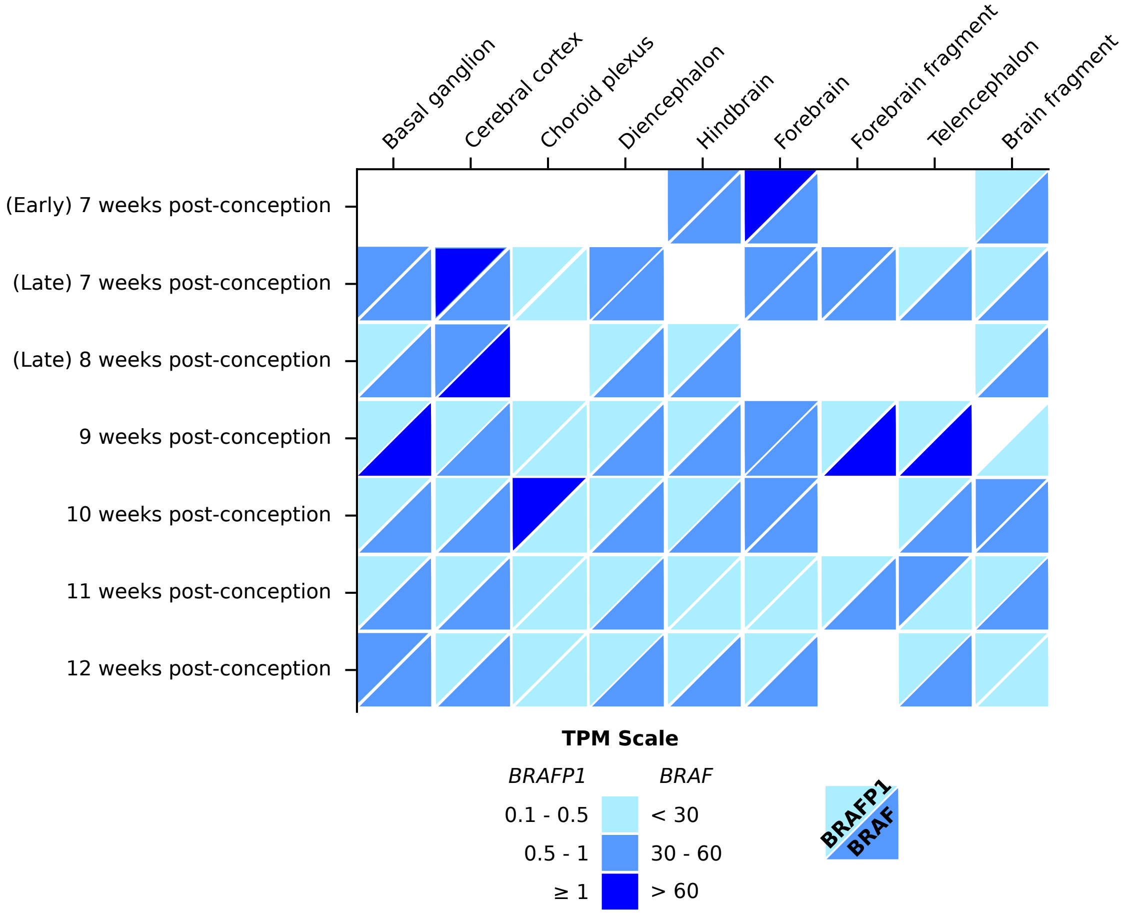

Supplementary Figure 4. Heatmap of *BRAF* and *BRAFP1* expression in human brain tissues during embryogenesis. RNA-seq-derived TPM data for different brain tissues and different stages of human embryogenesis were retrieved from the Human Brain Development Resource for both *BRAF* and *BRAFP1* (Gerrelli et al. 2015). In this resource, elements are considered expressed with a TPM ≥ 0.5, therefore we only include here tissues and stages which have *BRAFP1* expression ≥ 0.5 in at least one tissue/stage. However, we also display TPM levels between 0.1 and 0.5 for these samples, as *BRAFP1* is typically expressed in this range (Figure 3A-E). Blank squares indicate stages at which no data was available for that tissue.

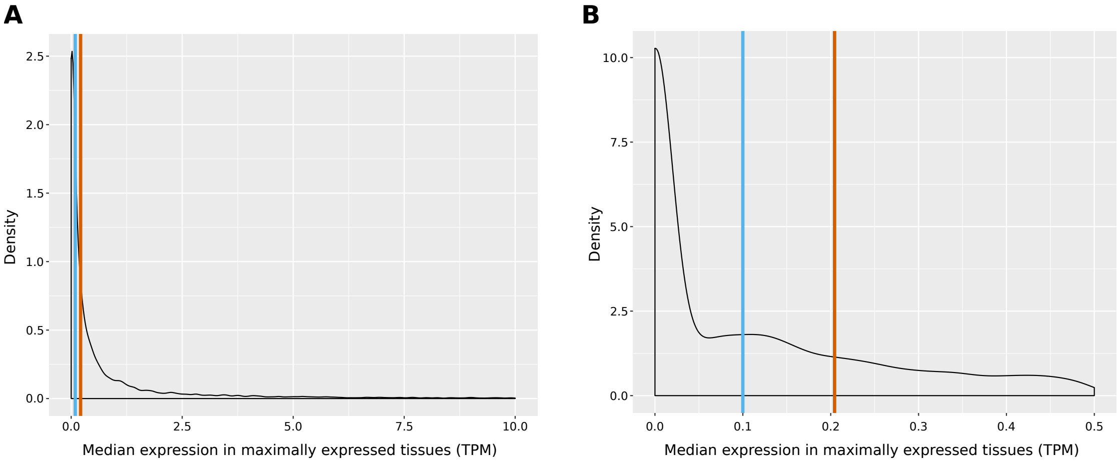

Supplementary Figure 5. Distribution of pseudogene median TPM values in their most highly expressed tissue. The density distributions of median expression values (TPM) between 0 and 10 **(A)** and 0 and 0.5 **(B)** are shown for pseudogenes in their most highly expressed tissue. The *BRAFP1* median expression level in the tissue in which it is most highly expressed is given by the red bar. To distinguish between expressed and non-expressed pseudogenes, by reducing the number of false positives and false negatives, we chose an expression cut-off of 0.1 TPM, shown as a blue bar on the distribution plots. In other words, if a pseudogene has a median TPM of ≥ 0.1 we consider it expressed. This level mirrors the expression rules outlined by the GTEx Consortium. However, our cut-off is slightly more conservative as we are requiring at least 50% of the samples in the tissue to have an expression value of 0.1 or greater (GTEx Consortium 2015).

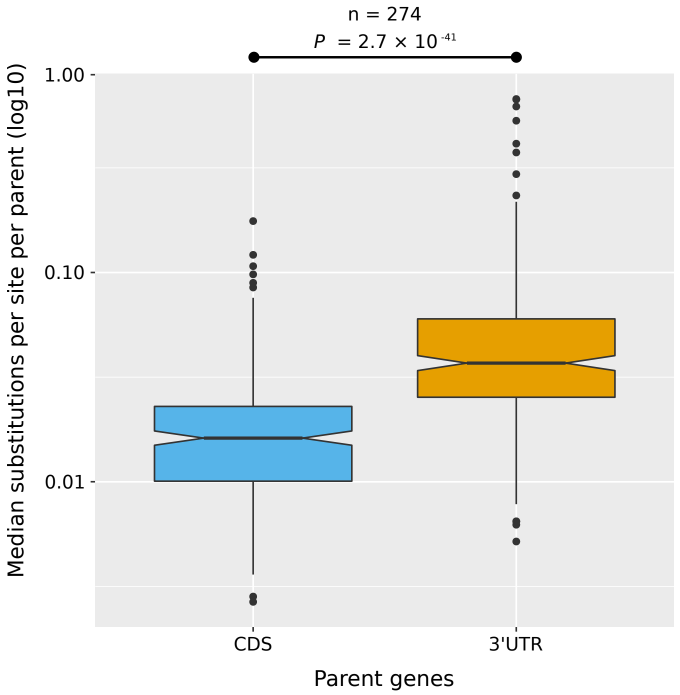

Supplementary Figure 6. Comparison of CDS regions and 3'UTRs of parent genes. Median number of substitutions in the CDS and 3'UTRs of 274 parent genes are shown. Only parent genes with one-to-one orthologs in at least 5 species since their respective pseudogene's formation were analysed, using the same alignment criteria as those for the pseudogene alignments. Bonferroni-corrected P-value was calculated by Wilcoxon signed-rank test.

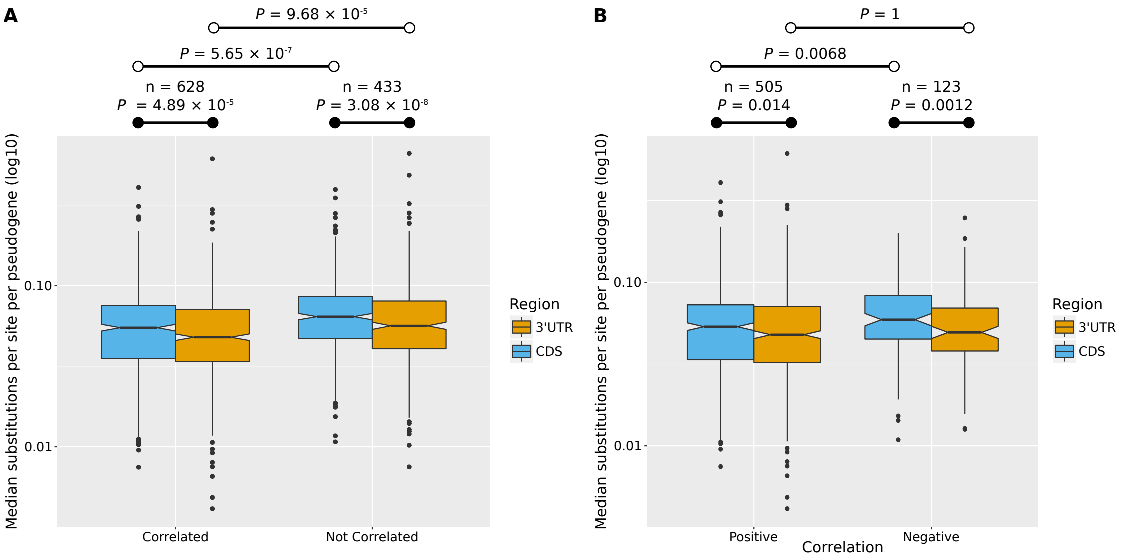

Supplementary Figure 7. Region substitution comparison of pseudogene and parent gene expression correlations. (A) Median number of substitutions for expressed pseudogenes that have correlation or no correlation with expression of their parent genes. Expression correlation was determined by Spearmann rank-order correlation test. For each pseudogene only tissues with both pseudogene and parent gene expression were analysed. Correlation P-values were Bonferroni-corrected for multiple testing. (**B**) Median number of substitutions for pseudogenes with positive or negative expression correlation with their parent genes. Bonferroni-corrected P-values for comparisons between different regions are calculated by Wilcoxon signed-rank test (●), and between same regions by Mann-Whitney U test (○).
